# Perovskite Thin Film Synthesised from Sputtered Lead Sulphide

**DOI:** 10.1038/s41598-018-19746-8

**Published:** 2018-01-24

**Authors:** José Maria Clemente da Silva Filho, Viktor A. Ermakov, Francisco Chagas Marques

**Affiliations:** Institute of Physics “Gleb Wataghin” – University of Campinas 777 Sérgio Buarque de Holanda Street - Cidade Universitária Zeferino Vaz, Barão Geraldo, Zip code: 13083-859 Campinas-SP Brazil

## Abstract

In the last few years, research on dye-sensitised devices has been focused on the development of solar cells, based on CH_3_NH_3_PbX_3_ (X = I^−^, Br^−^, Cl^−^) composites with perovskite structure. The deposition of perovskite thin films is usually carried out by solution-based processes using spin-coating techniques that result in the production of high quality films. Solar cells made by this method exceed 20% efficiency, with the potential for use in large scale production through ink print or screen printing techniques. As an alternative route, perovskite thin films can be deposited through thermal evaporation. A new method is proposed to produce CH_3_NH_3_PbI_3_, based on a radio-frequency (rf) -sputtering technique that results in a high reproducibility of the films and is compatible with roll-to-roll processes. We deposited thin films of lead-sulphide (PbS) and converted them into perovskite by placing the films in an iodine atmosphere, followed by dipping in a solution of methylammonium iodide (CH_3_NH_3_I). The conversions to PbI_2_ and CH_3_NH_3_PbI_3_ were confirmed by elemental analyses, absorption, and photoluminescence spectroscopy. Structural properties were revealed by X-ray diffraction and infrared and Raman spectroscopy.

## Introduction

Perovskite thin films based on organo-inorganic materials, such as CH_3_NH_3_PbX_3_ (X = I^−^, Br^−^, Cl^−^), have attracted the attention of researchers around the world in recent years due to their impressive optical and electronic properties^1,2^. These include a direct band-gap (1.4–3.0 eV)^3,4^, high absorption coefficient^5^, long charge carrier diffusion length^6^, and an ambipolar charge transport^7,8^. In addition, perovskite is a low-cost material that can be prepared on a large scale for mass production. The efficiency of perovskite solar cells increased significantly in a very short period of time, improving from 3.8% in 2009^9^ to 22.1% in 2016^10^, which is comparable with crystalline silicon solar cells^11–13^.

Perovskite thin films have been synthesised by adopting two precursors, *e*.*g*., lead iodide (PbI_2_) and methylammonium iodide (CH_3_NH_3_I) (also known as MAI), for CH_3_NH_3_PbI_3_ perovskites. They are deposited in two steps (one precursor at a time), or both precursors are mixed and deposited in a single-step process^14–16^. Several deposition process have been developed, such as spin-coating solution process^17,18^, doctor-blade^19^, vapor-assisted solution process, dual source evaporation process, and combinations of these techniques^20–22^. The one-step spin-coating technique was used in the first lead halide perovskite solar cells and consists of deposition of a mixture of PbI_2_ and CH_3_NH_3_I in a polar solvent, such as N,N-dimethyformide (DMF), followed by thermal annealing at 70–150 °C in order to crystallise the perovskite film^9,17,23^. However, some problems concerning stoichiometry and crystallinity control have arisen due to the uncompleted reaction of precursors in solution, and the annealing process for the one-step solution-processing method^24^. The two-step sequential solution-processing method provides a more controllable setup than the one-step method for perovskite deposition. The film quality can be improved through controlled crystal growth, which depends on the reaction time between PbI_2_ and CH_3_NH_3_I, and the post annealing duration^15,18,25,26^. Some approaches to solution engineering and controlled solvent evaporation have been used to improve the film quality and solar cell efficiency in the solution process deposition^14,26,27^. Nevertheless, the spin-coating technique (in one or two steps) has been associated with poor homogeneity and is incompatible with large-area and large-scale production^2,28^.

Thermal evaporation is another technique widely used to synthesise perovskite thin films^29,30^. This method is based on simultaneous thermal evaporation of perovskite precursors (lead-halide salt and CH_3_NH_3_I) in an evacuated chamber. The technique has been implemented using single or dual-source evaporation. Film deposited by thermal evaporation are more uniform and have a higher adherence to substrates than films deposited by spin-coating. Besides that, the films are also more compact and pinhole-free, making it compatible with planar solar cells^16,20,31^.

Some alternative approaches have been implemented to produce more compact and pinhole-free perovskite thin films. Among them, in a work by Sutherland *et al*.^32^, an atomic layer deposition (ALD) technique was used to deposit PbS as a seed layer for post-conversion into perovskite thin films, after exposure to iodide (I_2_ gas) and CH_3_NH_3_I vapour, respectively. Conformal deposition and atomic thickness precision are the main advantages of the ALD technique^33,34^. Even though perovskite thin films based on PbS films deposited by ALD exhibit many interesting qualities, the process involves an expensive apparatus, and it adopts dangerous precursors, i.e., H_2_S^35^.

In this work, we propose an alternative approach for producing planar perovskite thin films based on combination of rf-sputtering with the solution process route. PbS thin films with good homogeneity and adherence on bare glass and silicon have been successfully deposited by sputtering^36^. Thus, a new method is proposed to demonstrate that PbS thin films, deposited by rf-sputtering at room temperature, can be used as precursor films for post-conversion into perovskite (CH_3_NH_3_PbI_3_) thin films with good homogeneity and pinhole-free. This was achieved by converting the PbS thin films into PbI_2_ through exposure to iodide gas (I_2_) at room temperature, followed by dipping into a solution of MAI for a few minutes, and subsequently submitting them to annealing at 100 °C for 20 minutes. The procedure can be extended to the production of perovskite alloys (e.g., combinations of Pb, Sn, and Ge), adopting a composite target or the co-sputtering technique. More complex structures, such as those used in the manufacture of highly efficient solar cells^37,38^, with the inclusion of mixed cations (Cs, MA and FA) and halides (I, Br), seem to be also feasible. Since sputtering is a very well-known technique for thin films deposition^39–43^, and its application on industrial scale^42,44^ is already available in the market, this approach has potential to scale-up the perovskite solar cells production.

## Results and Discussion

### Structural and Morphological properties

**Figure 1:**
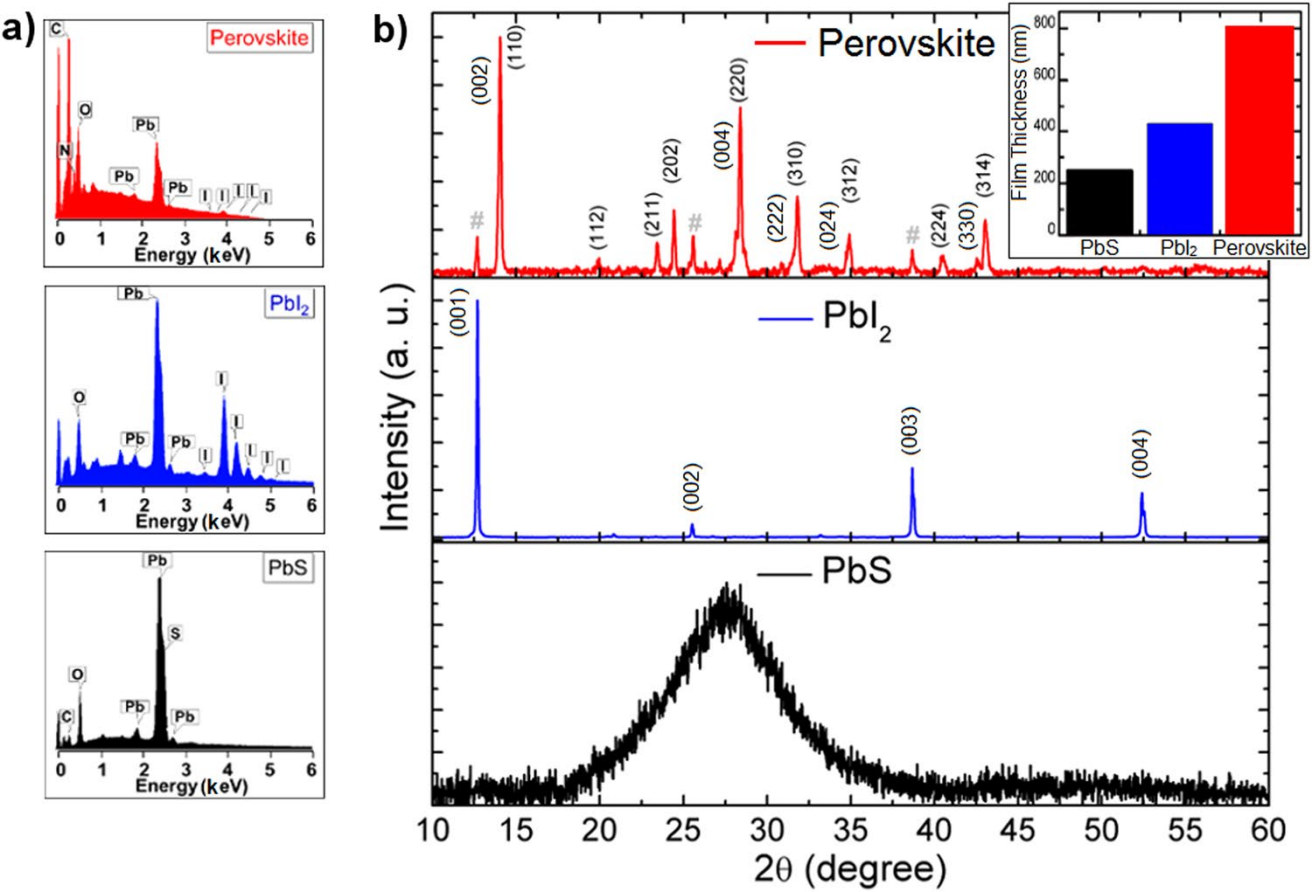
Elemental composition and structural analyses of films after each conversion step. (**a**) EDS and (**b**) XRD of films at each step. Remaining PbI_2_ are marked with #. The inset shows the thickness of films.

A PbS film 250 nm thick was completely converted into PbI_2_ through its exposure to an iodine atmosphere, generated by sublimation of solid iodine inside a closed petri plate. This step converts PbS into PbI_2_ by reaction with iodine: *PbS* + *I*_2_ → *PbI*_2_ + *S*(↑) ^45^. After the conversion the films were rinsed with ethanol to remove excess iodine. The resulting thickness of the PbI_2_ film was 430 nm (inset of Fig. 1b), approximately twice the thickness of the PbS. After conversion into perovskite, the final thickness was almost doubled again to approximately 800 nm. The increase in film thickness at each step is consistent with variation in the lattice parameters of PbS (*a* = *c* = 5.936 Å, JCPDS 02–0699), PbI_2_ (*a* = 4.557, *c* = 6.979 Å, JCPDS 07-0235), and CH_3_NH_3_PbI_3_ (*a* = 8.78 Å, *c* = 12.70 Å), since the volume of unit cell increases after each conversion stage, as discussed by Sutherland *et*. *al*.^32^.

The elemental analysis of the films performed by EDS, Fig. 1a, shows the presence of expected elements for each material, PbS, PbI_2_, and CH_3_NH_3_PbI_3_. Some small amounts of O were also detected, probably related to post-oxidation of the PbS film deposited at room temperature, and/or to adsorbed water.

**Figure 2:**
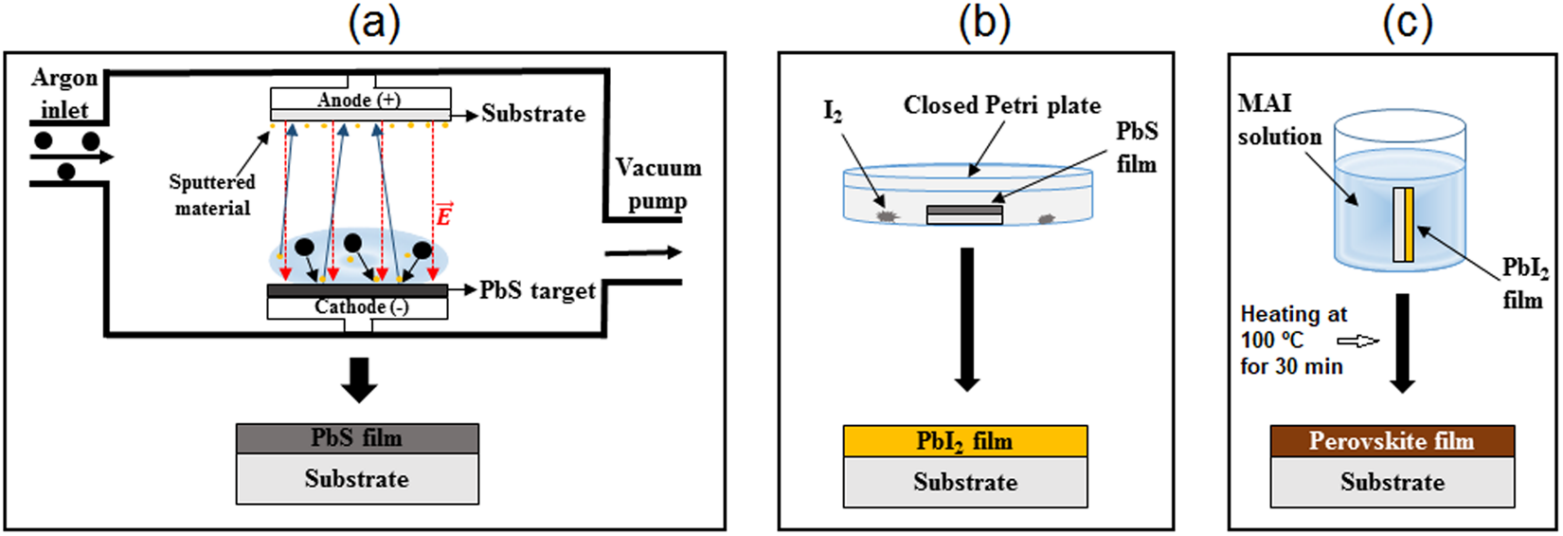
Schematic diagrams for the conversion of PbS into perovskite (CH_3_NH_3_PbI_3_): (**a**) PbS thin film deposited by sputtering on glass and Si substrates, (**b**) PbS film exposed to sublimated I_2_ for its conversion into PbI_2_, and (**c**) dipping the PbI_2_ film into MAI solution for conversion into perovskite.

XRD of the PbS film, deposited as shown in the Fig. 2a, reveals that it is completely amorphous without any signal from microcrystals (Fig. 1b), due to presence of just a wide band in the spectrum, which is in agreement with previously published results^36^. After treatment with I_2_ gas (Fig. 2b), the PbS film was completely converted into PbI_2_ (blue curve in Fig. 1b), as confirmed by standard PbI_2_ powder diffraction catalogued JCPDS 07-0235. The as-converted perovskite film (as shown in Fig. 2c) has XRD peaks corresponding to the planes (002), (110), (112), (211), (202), (004), (220), (222), (310), (024), (312), (224), (330) and (314), red curve in Fig. 1b. All these planes agree with previous published results^46,47^ and confirm the efficiency of our approach. Three peaks with low intensity and marked with # are associated with PbI_2_ crystals, which were not completely converted into perovskite. It is well known that the conversion of PbI_2_ into perovskite through post-treatment with MAI leaves a small percentage of unconverted PbI_2_. This has been attributed to the quick growth of a thick shell of perovskite when using the dipping technique in methylammonium iodide, hindering total conversion^48^. However, there is some debate in the literature about the detrimental effect of the remaining PbI_2_ in perovskite film, and some studies have reported even better devices if an appropriate amount of PbI_2_ is present^28,48–50^.

**Figure 3:**
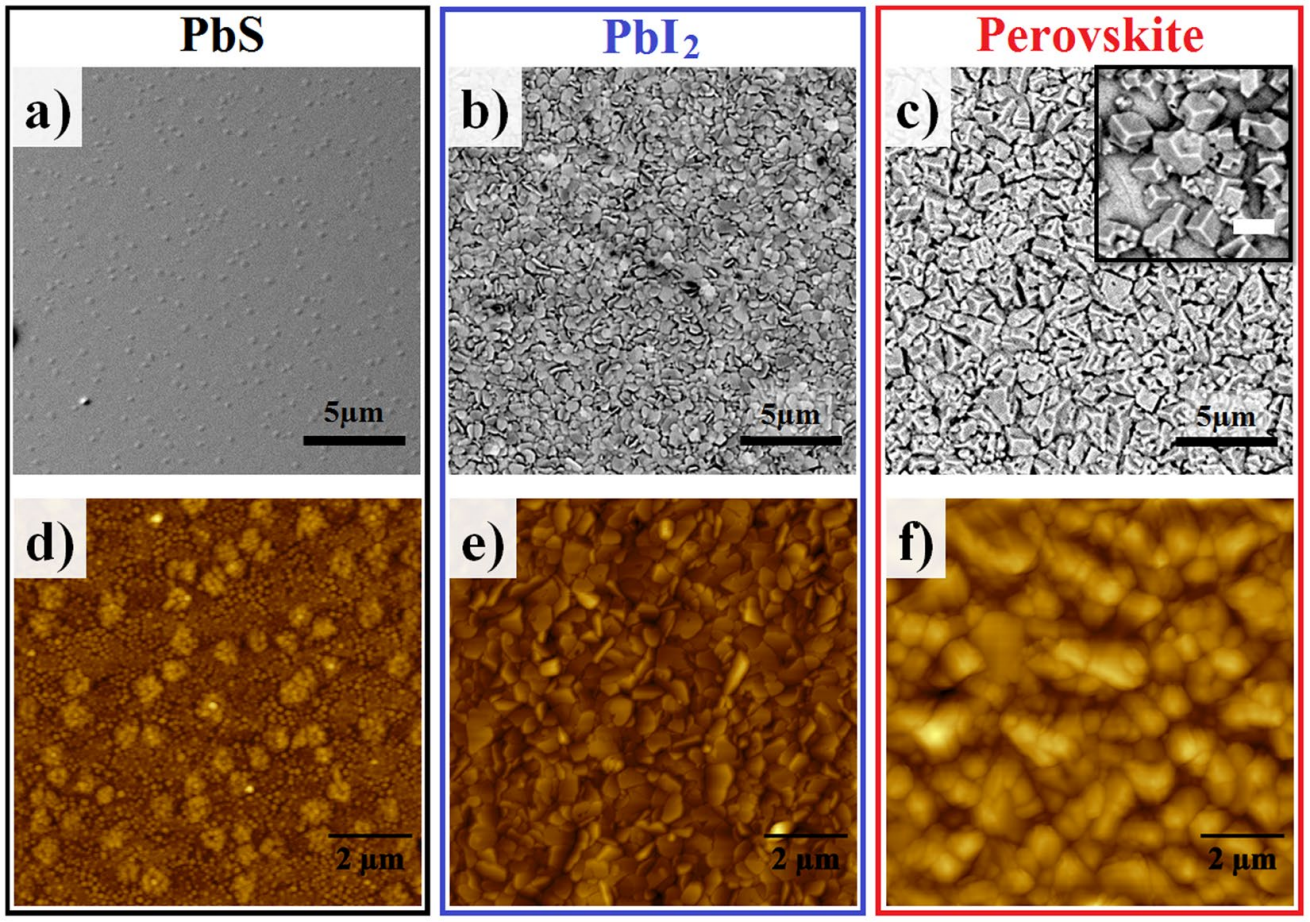
Morphological analyses of thin films. (**a**,**b** and **c**) are 20 × 20 µm^2^ SEM micrographs of PbS, PbI_2_, and CH_3_NH_3_PbI_3_ perovskite thin films, respectively. (**d**,**e** and **f**) are AFM micrographs in a 10 × 10 µm^2^ window of the same films. The inset on c) is a detail of the cuboid shape of a CH_3_NH_3_PbI_3_ perovskite crystal, the scale bar represents 2 µm.

Figure 3 shown typical SEM and AFM images of films. It can be seen from SEM images, that film morphology undergoes a substantial transformation after each conversion step. The grain size increases significantly, passing from small amorphous grains (~50 nm) on PbS (Fig. 3a) to crystalline pancake-shaped grains on PbI_2_ (Fig. 3b), and then to cuboid crystals on perovskite (Fig. 3c and inset).

Notable morphological variations also were seen through the AFM images, Fig. 3d–f. The increase in grain size was quantified through average roughness (R_a_) of the films, which was accessed by AFM measurements. The obtained values for R_a_ were 2.28 nm, 18.6 nm, and 71.1 nm, for PbS, PbI_2_, and perovskite films, respectively.

**Figure 4:**
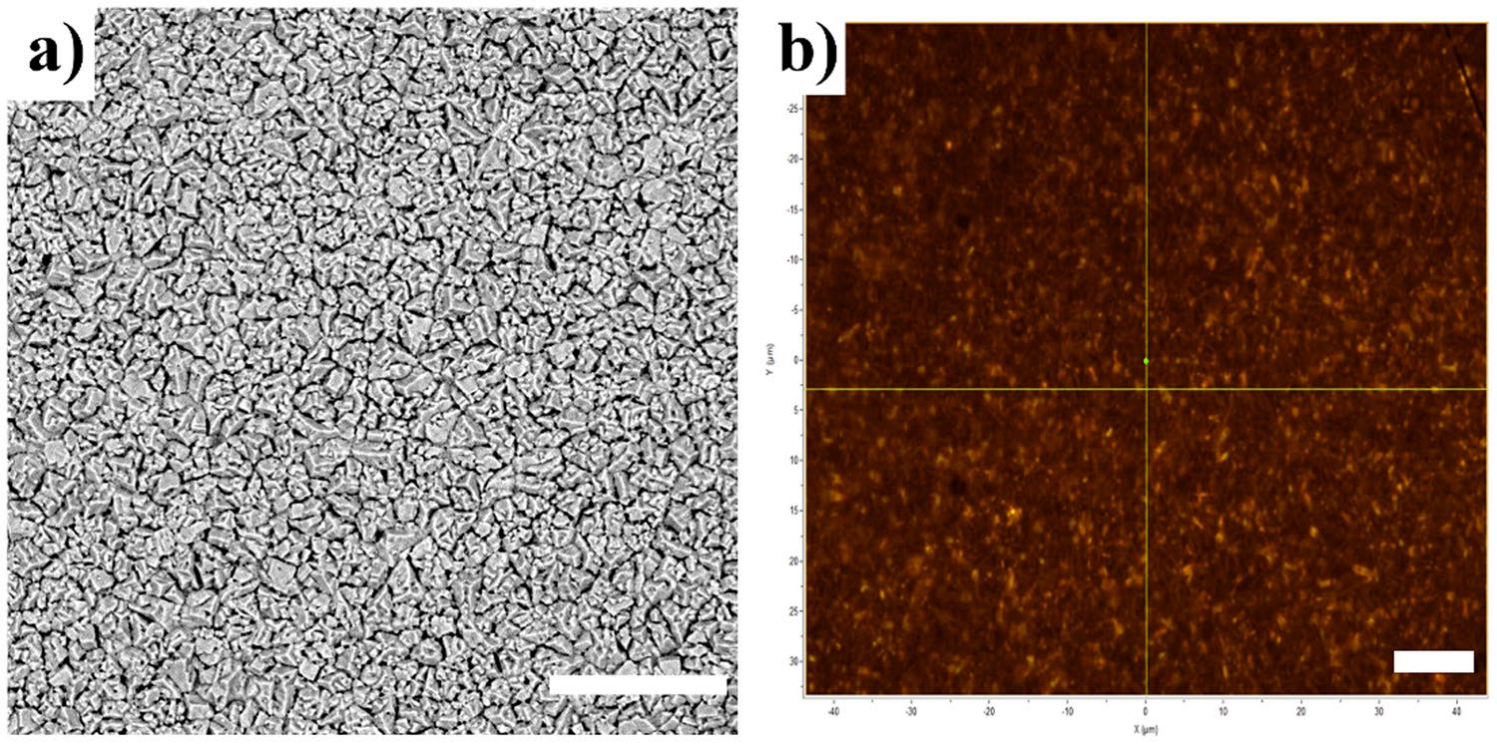
Large-area images of perovskite film. (**a**) (40 × 40µm^2^) SEM and (**b**) (100 × 70 µm^2^) optical. The scale bars represent 10 µm.

One of the main improvements proposed by this approach concerns to the complete substrate coverage and film homogeneity. To this end, we performed wide-area SEM (40 × 40 µm^2^) and optical (100 × 70 µm^2^) images from many points of the films and the Fig. 4 show typical images. It can be seen from the Fig. 4a that films present a homogenous crystal size distribution and connected crystals, which is essential to device production, besides, the films have no evidence of pinholes or cracks. The optical images, Fig. 4b, also confirm the quality of deposited films. In comparation with other popular techniques, like spin-coating, the films here synthesized presented higher surface uniformity and surface coverage.

### Vibrational and Optical Properties

**Figure 5:**
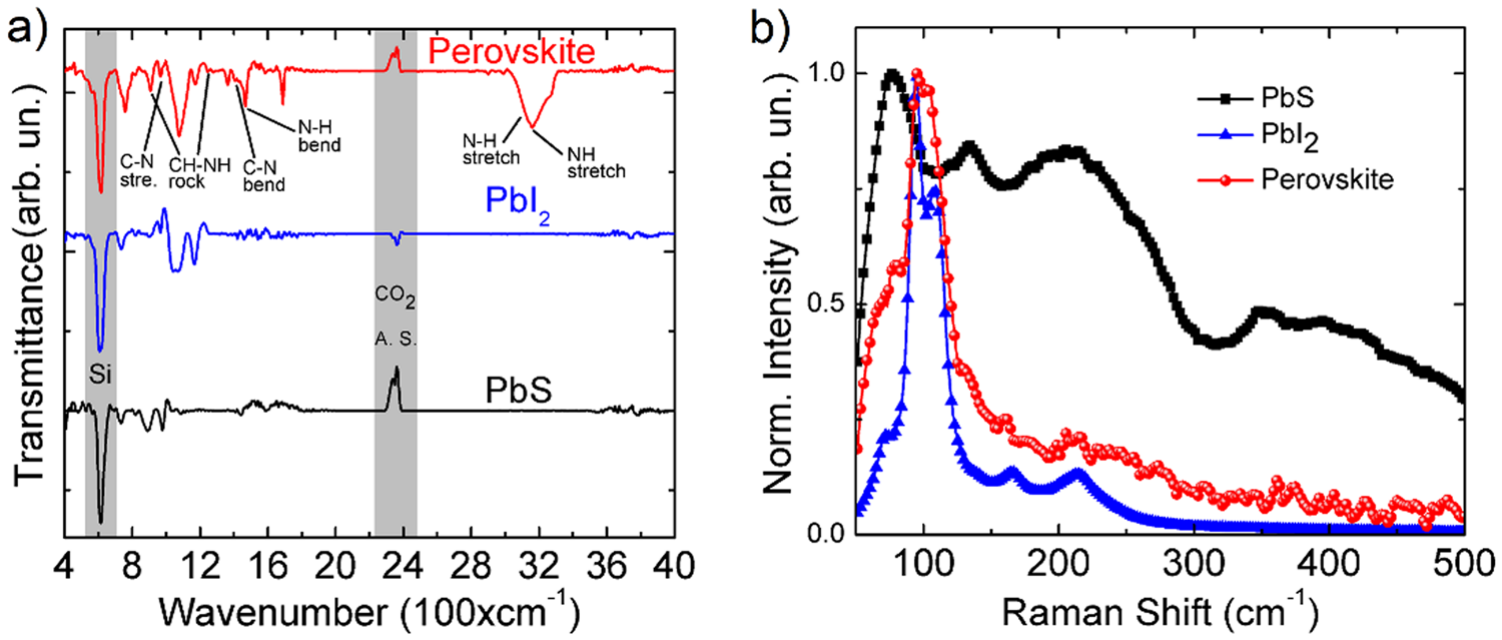
Vibration spectroscopy of films. (**a**) FTIR spectra in the wavenumber range 400 to 4000 cm^−1^ and (**b**) Raman in the 50 to 500 cm^−1^ wavenumber range.

Figure 5a reveals the FTIR spectral features of films in the wavenumber range 400–4000 cm^−1^. PbS and PbI_2_ have presented no IR peaks, since they are transparent in this region of wavelength. Just the asymmetric stretching of CO_2_ and a peak associated with the silicon substrate (indicated by vertical grey colour bars) are present in the spectra. Characteristic CH_3_NH_3_PbI_3_ IR peaks were detected in the range from 3000–3200 cm^−1^ (Fig. 5a), which were attributed to symmetric NH_3_^+^ and CH_3_ stretching modes. The absorption features in the 1400–1470 cm^−1^ range are assigned to symmetric NH_3_^+^ bending and asymmetric CH_3_ bending, and the CH_3_-NH_3_^+^ rocking and C-N stretching modes are in the 900–1250 cm^−1^ range, as shown in Fig. 5a. All of these peaks agree with results previously reported^51,52^ and demonstrate that the proposed conversion process is highly efficient.

The Raman spectroscopy for all films, Fig. 5b, was performed at room temperature and ambient conditions using the 532 nm excitation line of an argon laser. Aiming to avoid damage to the samples, a low laser power was applied in the measures (13 µW), which is four orders of magnitude lower than the total laser power.

Since PbS has a weak Raman signal, a precise measure is a challenger task, moreover using a low laser power. The expected Raman peaks for the PbS should be at 154, 205 and 454 cm^−1^ ^53,54^. However, due to low scattering efficiency for excitation with 532 nm (2.33 eV), just a broad band centred at 206 cm^−1^ was detected, which has been assigned to first order longitudinal-optical (LO) phonons^53,54^. Since the PbS thin film were kept under room conditions and its surface is very sensitive to oxidation, the Raman spectrum of PbS also show bands at 77, 134 cm^−1^, which were attribute to surface oxidation (PbO and PbSO_4_)^54,55^. The wide shoulder in the range of 300–450 cm^−1^ is accounted to the glass substrate.

The Raman spectrum of the as converted PbI_2_ shows bands at 72, 94, 109, 165 and 214 cm^−1^ (blue curve in the Fig. 5b). As discussed elsewhere^56–59^ the peaks at 72, 94, 109, 165 cm^−1^ are assigned to the four allowed LO and traverse optical phonon (TO) modes ${E}_{2}^{1}(TO),{A}_{1}^{1}(TO),{A}_{1}^{2}(LO)$ and $2{E}_{2}^{1}(TO)$, respectively. The band at 214 cm^−1^ is attributed to the overtone of the 109 cm^−1^ band^60^. These results also indicate that PbS was completely converted into PbI_2_ after treatment with iodine.

The Raman spectrum of the perovskite film (red curve in the Fig. 5b) shows some features at 75, 90, and 105 cm^−1^, followed by some structures in the 105–500 cm^−1^ range which are probably associated with noise since the measurements were performed at a very low power to avoid artifacts. The origin of Raman signals from perovskite films are still under debate, since the few reported studies are contradictory or not well understood. The problem is related to degradation due to laser heating^61^. Quarti *et al*.^62^ and Park *et al*.^63^ attributed peaks above to the vibrational modes of inorganic octahedral PbI_6_, which is part of the perovskite structure, based on comparison between density functional theory (DFT)-simulated Raman spectra and experimental results.

**Figure 6:**
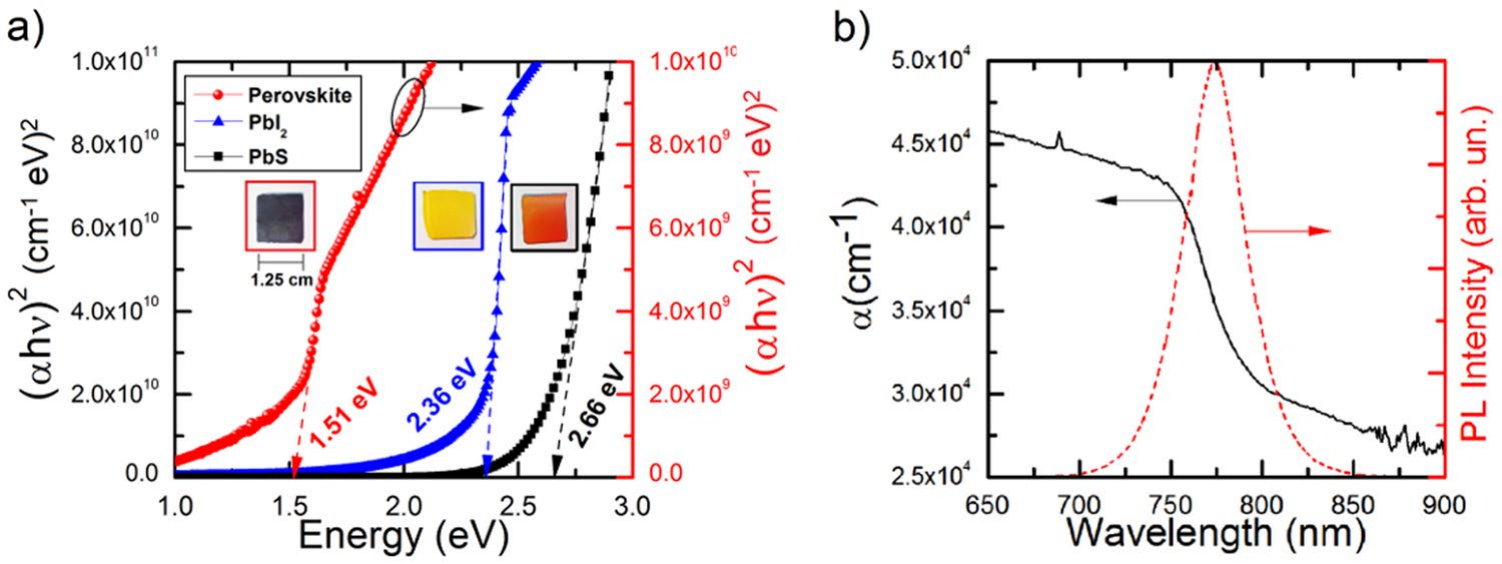
Band-gap calculation for the films at each step. (**a**) Tauc’s plot (where α = absorption coefficient, h = Plank’s constant and ν = frequency) indicating the band-gap of the film (dashed lines). The insets are pictures of the samples after each conversion step. (**b**) Absorption and photoluminescence of the perovskite film as a function of wavelength.

Interesting results were obtained from optical measures for each conversion step, Fig. 6. Aiming to get good optical measurements, the deposition time for PbS thin films was 360 minutes, which produced films of approximately 750 nm. Thus, the thickness of PbI_2_ and perovskite films after the conversion becomes 1.3 and 2.3 µm, respectively. The band gap of deposited films was determined using the Tauc’s relation, Equation (1)^64^:

1 $$\alpha h\nu =C{(h\nu -{E}_{g})}^{n},$$

where *α* is the absorption coefficient, *E*_*g*_ is the band gap, *hν* is the incident photon energy and *C* is a constant.

In the range of high absorption coefficient (*α* > 10^4^* cm*^−1^), the band gap of PbS films was 2.66 eV (Fig. 6a). This is a very wide band gap considering that monocrystalline PbS has a band gap of only 0.41 eV^60^. The reason for this sizeable discrepancy is because our films were deposited at room temperature and are amorphous (black curve in the Fig. 1b), which promote tail states in the band gap onset. However, the obtained results confirm to what has previously been reported in the literature^36,65^. From our own experience, PbS Films deposited at 300 °C were crystalline with a band gap of 0.72 eV (not shown here).

After the conversion of amorphous PbS into PbI_2_ is possible to see a huge change in the film colour, which change from semi-transparent to completely yellow (insets in Fig. 6a). The colour change is associated with a decrease in the band gap, which was reduced from 2.66 to 2.36 eV, the expected value for this material^66^. This reduction in the band gap reinforces the results obtained from XRD measurements about the complete structural transformation from PbS to PbI_2_.

After the conversion to perovskite, the films experienced another decrease in the band gap, attaining 1.51 eV^13,27,67^. This optical change can be easily seen by change in the film colour, that now went from yellow to dark brown (insets in Fig. 6a). The photoluminescence spectrum of the perovskite film at room temperature (Fig. 6b) showed a peak centred at 780 nm (~1.58 eV), which is located at the onset of the absorption coefficient attributed to the band gap, also in agreement with previous reports^12^. Thus, these optical measurements showed that this approach produce perovskite thin films with appropriate optical characteristics for solar cells application, as previously proposed.

## Conclusions

In summary, we demonstrated a new method to produce perovskite films using sputtering. At the first step, an amorphous PbS thin film is deposited, which is then converted into PbI_2_ through treatment with iodine. The PbI_2_ film was subsequently converted into a CH_3_NH_3_PbI_3_ perovskite film by dipping it into methylammonium iodide. The complete process was performed at a temperature less than 100 °C, allowing its use in a great variety of substrates. The efficiency of the conversion processes from PbS to PbI_2_ and finally to CH_3_NH_3_PbI_3_ was demonstrated through chemical analyses using EDS, photoluminescence, X-ray diffraction, FTIR, and Raman spectroscopy. The main advantages introduced by this method were the production of perovskite films with high surface uniformity over large areas and films with high optical absorption on visible spectrum. Besides, this route can act as an alternative to the conventional chemical bench approach, since sputtering is an easy, cost-effective, and well-known technique for thin film deposition. Additionally, its implementation in large-area and large-scale production is already consolidated in the electronic market.

## Methods

### Sample preparation

The perovskite films were deposited in three steps, as shown in Fig. 2. Prior to deposition, the substrates were sonicated in a soap solution in water, acetone, and ethanol, for 15 minutes each. The cleaning process was finished with UV-ozone treatment for 30 minutes at 70 °C, to remove any organic contamination. The base pressure for deposition was 4 × 10^−6^ mbar. Firstly, a PbS target (Stanford Advanced Materials) of 99.9% purity was sputtered in a rf-sputtering system (Leybold-Heraus Z400) at a bias of −1000 V, under argon (99.99999% purity) pressure of 3 × 10^−3^ mbar (Fig. 2a). All films were deposited on double-side polished crystalline silicon and glass substrates (1.25 × 1.25 cm^2^) at room temperature. The deposition rate was approximately 2.0 nm/min, and there was no intentional heating during the deposition time, which were 120 and 360 min. However, during the deposition the temperature spontaneously reached approximately 50 °C.

The conversion of the PbS films into perovskites was done in two steps. First, the PbS films were placed inside a sealed enclosure together with approximately 100 mg of solid iodine (I_2_) (Sigma-Aldrich) with 99.99% purity. Due to the natural sublimation process at room temperature, a vapor pressure of I_2_ is established inside the container. It reacts with the PbS film (Fig. 2b), converting it into PbI_2_ after 60 hours, as probed through X-ray diffraction (XRD). This conversion can be catalyzed by temperature, which would reduce the reaction time, but in this report, we will carry out the reaction at room temperature. The last step consisted of dipping the PbI_2_ thin film into a solution of 10 mg/ml of MAI in isopropyl alcohol heated to 50 °C for 5 minutes, as shown in Fig. 2c. After conversion, the film was annealed at 100 °C, for 30 minutes, in an open furnace. During all synthesis, the relative humidity was in the 50 to 70% range.

### Characterization

Optical properties were extracted from a UV/VIS/NIR Spectrophotometer (Lambda 9, Perkin-Elmer) in the wavelength range 190 to 3200 nm (6.53 to 0.39 eV). Vibrational properties were obtained using Fourier transform infrared spectroscopy (FTIR) in transmittance mode, in the wavenumber range 400 to 4000 cm^−1^ (Jasco 6100). Raman scattering was carried out in a confocal micro-Raman configuration (XploRA, Horiba), in the wavenumber range 50 to 500 cm^−1^. XRD was performed using a D8 Advanced system (Bruker) equipped with a Cu*Kα* (0.15418 nm) X-ray source, operating at 40 kV and 40 mA. The Bragg–Brentano θ–2θ configuration was used, with incidence angle of 1°, an integration time of 10 s, and a step of 0.02°. Atomic force microscopy (AFM) images were obtained for superficial analysis (easyScan 2 Flex, nanoSurf) using a large area (100 × 100 µm^2^) scanner in *tapping* mode. The tip radius was smaller than 10 nm. Surface morphology measurements were also performed using Scanning Electron Microscopy (SEM) (Phenon, FEI), with an accelerating voltage of 5 kV in secondary electron scattering mode. For these analyses, the samples were deposited on double-sided polished c-silicon wafers. Energy-dispersive X-ray spectroscopy (EDS) was performed for elemental analyses using a FIB-SEM Nova 200 Nanolab system from FEI, with 10 kV accelerating voltage. The thickness of the films was measured in a DEKTAK 150 Profilometer by Veeco. All measurements were performed immediately after the films were prepared, as they degrade with exposure to moisture, similar to those prepared by solution processes.
